# Social inequality and diabetes mellitus – developments over time among the adult population in Germany

**DOI:** 10.25646/5986

**Published:** 2019-06-27

**Authors:** Christin Heidemann, Yong Du, Jens Baumert, Rebecca Paprott, Thomas Lampert, Christa Scheidt-Nave

**Affiliations:** Robert Koch Institute, Berlin Department of Epidemiology and Health Monitoring

**Keywords:** DIABETES MELLITUS, EDUCATION, PREVALENCE, DIABETES RISK, DIABETES CARE

## Abstract

The connection between social disadvantage and the presence of known diabetes and specific risk factors is well documented. This article summarises the results from the Robert Koch Institute examination surveys that were conducted between 1997 and 1999 as well as 2008 and 2011 to address social inequality – operationalised by level of education – with regard to prevalences of known and unknown diabetes, risk of diabetes and care of diabetes as well as their development over time. Both survey periods showed that the low education group has higher prevalences of known and unkown diabetes as well as a higher risk of developing diabetes within the next five years compared to the medium and high education group. Over time, prevalence tended to increase for known diabetes and to decrease for unknown diabetes for all education groups. For the 5-year diabetes risk, only the high education group showed a clear decrease over time. The chosen indicators of diabetes care indicated no clear differences between education groups and an improvement of diabetes care over time. For some indicators of care (foot examination, statins), improvements were only seen in the low education group. In conclusion, social inequalities in the prevalence of known and unknown diabetes as well as in diabetes risk remain in Germany; for the indicators of care, however, no clear education gradient is evident. Over time, inequality regarding the prevalence of diabetes has not increased further. However, with regard to diabetes risk, inequality has become slightly more evident. For individual care indicators, improvements are limited to specific education groups.

## 1. Introduction

Diabetes mellitus is a metabolic disease with a disturbed regulation of blood glucose (blood sugar) [1]. The main risk factors of the disease’s most common form, type 2 diabetes, include unfavourable health-relevant behaviours such as lack of exercise, an unhealthy diet, and smoking, along with being overweight as a frequent consequence [2]. Unknown or inadequately treated diabetes leads to chronically elevated blood glucose concentrations. Blood vessels and the nervous system can be subsequently damaged, resulting in diabetes-specific complications (such as renal dysfunction, eye disease, diabetic foot syndrome, and amputations of the lower limb) as well as cardiovascular diseases (such as heart attack and stroke). These severe diabetes-related diseases lead to a loss of health-related quality of life and life expectancy for affected individuals and place a high financial burden on the health system [3]. Due to these health impacts and the rising prevalence of diabetes – particularly of type 2 diabetes, which tends to become more frequent with increasing age – diabetes presents a major public health challenge in Germany and most other countries [2, 4].

**Info box 1:**

**Nationally representative interview and examination surveys for adults conducted by the Robert Koch Institute (cross-sectional surveys)**

**►** German National Health Interview and Examination Survey 1998 (GNHIES98, 1997-1999)
**►** German Health Interview and Examination Survey for Adults (DEGS1, 2008-2011)

**Objectives:** Providing information on health status, health behaviour and health care of the population in Germany and analysis of trends over time

**Survey methods:** Self-administered written questionnaire, physical examinations and tests, computer-assisted medical interview, assessment of currently used medications, laboratory analyses of blood and urine samples

**Target population:** Adults aged 18 to 79 years with permanent residence in Germany

**Sampling method:** GNHIES98: Registry office sample; randomly selected individuals from 120 municipalities in Germany were invited to participate DEGS1: Registry office sample; randomly selected individuals from 180 municipalities in Germany were invited to participate (120 original sample points of the GNHIES98 and 60 new sample points).

**Participants:** GNHIES98: 7,124 adults

DEGS1: 7,987 adults (3,795 GNHIES98 revisiting participants, 4,192 first time participants)

**Response rate:** GNHIES98: 61%

DEGS1: 64% for GNHIES98 revisiting participants, 42% for first time participants

More information can be found at Thefeld et al. 1999 [8], Kamtsiuris et al. 2013 [9] and Scheidt-Nave et al. 2012 [10] as well as at www.degs-studie.de/en

Analyses of the temporal development of the presence of diabetes and its risk factors as well as the diabetes care situation are a decisive prerequisite to adapt or monitor prevention and care measures and to estimate the development of diabetes-related health system expenditure. As socioeconomic factors are often related to health-relevant behaviour and its associated diseases, comparing population groups and, where appropriate, developing target group- or setting-specific measures are important [5].

Such an analysis and provision of results on risks, presence and care of diabetes in Germany is the purpose of the national diabetes surveillance currently being developed at the Robert Koch Institute (RKI) [6]. The aim is to regularly provide information on 40 defined indicators or indicator groups [7]. This article considers the development over time of some key indicators of diabetes surveillance from the fields of diabetes risk, diabetes prevalence (frequency of diabetes) and diabetes treatment for the overall German adult population as well as stratified by level of education and gender. It is based on the data of the population-representative health interview and examination surveys of the RKI.

## 2. Methodology

### 2.1 Study population

**Figure 1:**
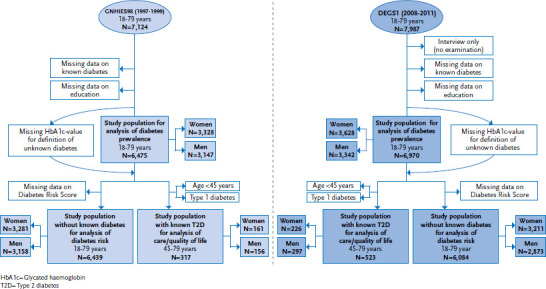
Study populations of the German National Health Interview and Examination Survey 1998 and the German Health Interview and Examination Survey for Adults Source: GNHIES98 (1997-1999), DEGS1 (2008-2011)

This article is based on data collected by two nationwide interview and examination surveys, the German National Health Interview and Examination Survey 1998 (GNHIES98, 1997-1999) and the German Health Interview and Examination Survey for Adults (DEGS1, 2008-2011) conducted as part of the continuous health monitoring at the RKI (Info box 1). Figure 1 shows the study populations as they were defined for both survey periods and used for the estimation of indicators of diabetes prevalence, diabetes risk and diabetes care.

### 2.2 Indicators

The definitions can be found for the indicators known diabetes and unknown diabetes in info box 2, for the indicator 5-year risk of developing type 2 diabetes in info box 3, for indicators related to the quality of care for type 2 diabetes in the areas of achieving treatment goals and self-management or medical care in info box 4 and info box 5, and for subjectively assessed health-related quality of life of type 2 diabetes in info box 6.

The data used to calculate indicators stem from GNHIES98 and DEGS1 and were collected in the form of self-administered written questionnaires (including questions on smoking habits, physical activity, diet, health-related quality of life), physical examinations (including measurements of body height, waist circumference, systolic and diastolic blood pressure), a computer-assisted medical interview (including questions on physician-diagnosed diseases, family history of diabetes, self-monitoring of blood glucose, medical examinations of the eyes and feet), an assessment of currently used medications (including documentation of medications to treat diabetes (anti-diabetic drugs) and certain medications to treat lipid metabolism disorders (statins)) as well as laboratory analyses of blood samples (including determination of glycated haemoglobin (HbA1c, a long-term blood sugar value), total and high-density lipoprotein cholesterol (HDL cholesterol)). In both surveys, indicators were obtained by similar methods. Only the health-related quality of life was assessed by different versions of the Short Form-36 Health Survey (SF-36). However, a recommended standardisation procedure was used to ensure comparability between the two versions [11]. The family history of diabetes was assessed exclusively in DEGS1. Detailed descriptions of the data collection process have been published elsewhere [11-14].

**Info box 2:**

**Indicator known diabetes:**

**►** Physician-diagnosed diabetes mellitus or
**►** Taking anti-diabetic drugs (ATC-Code A10)

**Indicator unknown diabetes:**

**►** No known diabetes and
**►** Glycated haemoglobin (HbA1c) value ≥6.5%

Both indicators are given as a prevalence, i.e. a proportion (in %) of people with known or unknown diabetes mellitus (without differentiating between types of diabetes) in the population.

Source: Nationale Diabetes-Surveillance am Robert Koch-Institut 2018 [7], Heidemann et al. 2016 [12]

ATC = Anatomical Therapeutic Chemical Classification System

All indicators are stratified by level of education and, additionally, gender. Level of education as an indicator of social inequality was defined via the CASMIN Index (Comparative Analysis of Social Mobility in Industrial Nations). This is based on the data provided by self-administered written questionnaires in GNHIES98 and DEGS1, which take both general and vocational training into account and allow a categorisation into low, medium and high education groups [15]. To depict indicators of care among persons with type 2 diabetes, the medium and high education group were combined to avoid that the number of cases in the subgroups become too small.

**Info box 3:**

**Indicator 5-year risk of developing type 2 diabetes**

The German Diabetes Risk Score (DRS) developed by the German Institute of Human Nutrition Potsdam-Rehbrücke calculates the absolute 5-year risk of developing type 2 diabetes (in %) for people who have so far not been diagnosed with diabetes. For example, a 5-year risk of 8% means that eight out of 100 people with the same DRS points will be diagnosed with type 2 diabetes over the course of the next five years.

**Diabetes Risk Score (DRS) points =**

5,1 x age (years)
+7.6 x waist circumference (cm)
–2.7 x body height (cm)
+47 x physician-diagnosed hypertension
–2 x physical activity (hours/week)
+15 x former smoker <20 cigarettes/day
+45 x former smoker ≥20 cigarettes/day
+23 x smoker <20 cigarettes/day
+77 x smoker ≥20 cigarettes/day
–7 x whole grain consumption
(per 50g portion/day)
– 5 x coffee consumption (per 150ml cup/day)
+ 55 x red meat consumption
(per 150g portion/day)
+ 56 x 1 parent with diabetes
+ 106 x both parents with diabetes
+ 48 x brother or sister with diabetes

**5-year risk of developing type 2 diabetes =**

1 – 0.99061 exp [(DRS points – 474.17096591)/100]

This article indicates the average 5-year risk of developing diabetes for the population without known diabetes.

Source: Nationale Diabetes-Surveillance am Robert Koch-Institut 2018 [7], Paprott et al. 2017 [13]

### 2.3 Statistical analysis

All prevalence estimates and mean values as well as corresponding 95% confidence intervals have been calculated using weighting factors. Weighting factors correct for deviations within the sample from the population structure as of 31 December 1997 for GNHIES98 and 31 December 2010 for DEGS1 (regarding sex, age, region, German citizenship, size of municipality and education) and also consider the deviations in probability of participation in DEGS1 between participants who previously took part in GNHIES98 and DEGS1 first time participants [8-10]. For a comparison over time of the total samples of GNHIES98 and DEGS1 that was independent of the changes in the age pyramid, GNHIES98 data were age-standardised based on the population as of 31 December 2010. To take into account both weighting and the correlation of participants within one municipality, results were calculated using SAS 9.4 survey procedures. Differences with p-values <0.05 were considered as statistically significant.

## 3. Results

### 3.1 Prevalence of known and unknown diabetes

**Figure 2:**
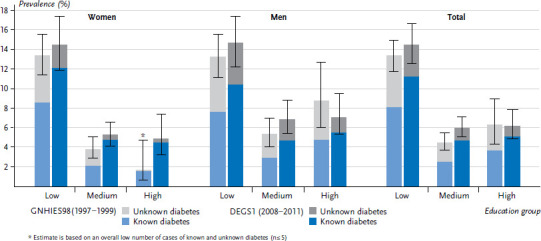
Prevalence of known and unknown diabetes over time for the 18- to 79-year-old population according to gender and education level (GNHIES98 n=3,328 women, n=3,147 men; DEGS1 n=3,628 women, n=3,342 men) Source: GNHIES98 (1997-1999), DEGS1 (2008-2011), Heidemann et al. 2016 [12]

To reflect the total diabetes burden in the population, the diabetes surveillance provides data for both indicators the prevalence of known diabetes as well as the prevalence of unknown diabetes (Info box 2). For the survey periods 1997 to 1999 and 2008 to 2011, the prevalence of known diabetes was 5.6% and 7.2%, the prevalence of unknown diabetes was 3.8% and 2.0%, and the prevalence of total diabetes was 9.3% and 9.2%,respectively. Once differentiated by level of education, higher prevalence estimates of known and unknown diabetes, and therefore also of total diabetes, were found in the low compared to the medium and high education group in both survey periods. For the period between the two surveys, tendencies towards an increase in the prevalence of known diabetes and a decrease in the prevalence of unknown diabetes were evident in all education groups. For overall diabetes, no significant changes in prevalence over time were observed [12] (Figure 2).

The sex-stratified analysis showed no significant differences in the prevalence of known diabetes between women (1997-1999: 5.7%, 2008-2011: 7.4%) and men (5.5%, 7.0%) [12]. Men, however, had a higher prevalence of unknown diabetes (1997-1999: 4.3%, 2008-2011: 2.9%) compared to women (3.2%, 1.2%) [12]. During the first survey period, this difference was particularly clear for the high education group, and during the second period, it was apparent for all education groups. Overall, however, for both survey periods, the pattern of higher prevalence estimates of known and unknown diabetes in the low education group compared to the medium and high education groups has been observed for both genders. Over time, the prevalence of known diabetes increased and the prevalence of unknown diabetes decreased for both genders [12], whereby a further differentiation by education group was not always permitting robust findings due to the low number of diabetes cases for certain subgroups – this applies in particular to women with high education in the survey period 1997 to 1999 (Figure 2).

**Info box 4:**

**Indicators of quality of care for type 2 diabetes – achieving treatment goals**

**Treatment goal for HbA1c**

**►** In the presence of diabetes-specific complications (diabetic renal dysfunction, diabetic eye disease, diabetic neuropathy, diabetic foot, diabetes-related amputation) or cardiovascular comorbidity (physician-diagnosed stroke, heart insufficiency, heart attack or other coronary heart diseases) and an age ≥45 years: HbA1c value <8.0%
**►** In the absence of diabetes- specific complications and cardiovascular comorbidity:
  **►** For an age ≥65 years: HbA1c value <7.5%
  **►** For an age 45 to 64 years: HbA1c value <7.0%

**Treatment goal for non-HDL cholesterol:**

**►** Total cholesterol serum value minus HDL cholesterol serum value <130 mg/dl

**Treatment goal for blood pressure:**

**►** Systolic blood pressure <140 mmHg and
**►** Diastolic blood pressure <80 mmHg

Each indicator is given as proportion (in %) of persons with type 2 diabetes who achieve the respective treatment goal in relation to all persons with type 2 diabetes.

Source: Nationale Diabetes-Surveillance am Robert Koch-Institut 2018 [7], Du et al. 2015 [14]

HbA1c = glycated haemoglobin (long-term blood sugar value) non-HDL = non-high-density lipoprotein

### 3.2 5-year risk of developing type 2 diabetes

**Figure 3:**
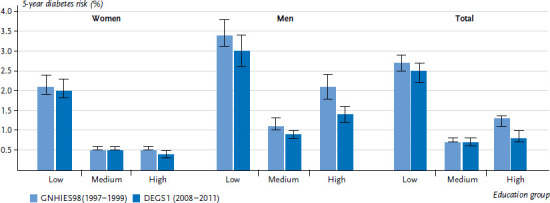
5-year risk of developing type 2 diabetes over time for the 18- to 79-year-old population without known diabetes according to gender and education level (GNHIES98 n=3,281 women, n=3,158 men; DEGS1 n=3,211 women, n=2,873 men) Source: GNHIES98 (1997-1999), DEGS1 (2008 –2011), Paprott et al. 2017 [13]

The indicator for the 5-year diabetes risk combines a set of established diabetes risk factors and helps to estimate how likely it is that a person will be diagnosed with type 2 diabetes within the next five years (Info box 3). For the survey periods 1997 to 1999 and 2008 to 2011, the average 5-year diabetes risk for the population was 1.5% and 1.1%, respectively. For both survey periods, the risk of getting diagnosed with diabetes was significantly higher for the low education group compared to the medium and high education groups. Over time, a significant decrease in the 5-year diabetes risk was only observed in the high education group [13] (Figure 3).

Stratified by gender, the analysis of both survey periods indicated a higher 5-year diabetes risk for men (1997-1999: 2.2%, 2008-2011: 1.5%) relative to women (1.1%, 0.8%). This difference between genders was observed across all education groups. In both genders, the pattern of a higher 5-year diabetes risk for the low education group compared to the medium and high education groups was evident. While for women, only the high education group showed a decrease in the 5-year diabetes risk over time, this tendency was visible across all education groups for men. However, it was also most pronounced in the high education group among men (Figure 3).

### 3.3 Type 2 diabetes care

#### Achieving treatment goals

The indicators for treatment goals of HbA1c-, non-HDL cholesterol and blood pressure were defined in the context of the diabetes surveillance based on treatment targets set out in the national guideline for the therapy of type 2 diabetes [16] or in the international recommendations on the treatment of high blood glucose levels [17, 18] and lipid metabolism disorders in people with type 2 diabetes [19, 20] (Info box 4). By outlining the proportion of persons with type 2 diabetes who achieve the respective treatment goal, they provide intermediate outcome measures on the quality of care for type 2 diabetes.

**Figure 4:**
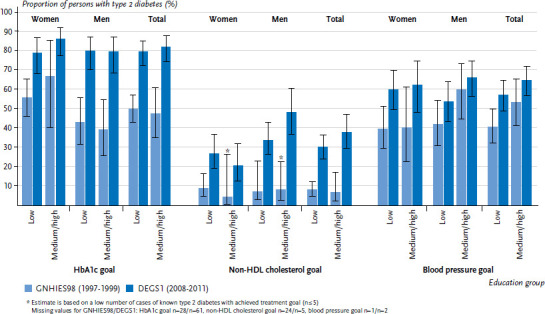
Comparison over time of the proportion of 45- to 79-year-old persons with type 2 diabetes who reach the treatment goal for HbA1c, non-HDL cholesterol or blood pressure according to gender and education level (GNHIES98 n=161 women, n=156 men; DEGS1 n=226 women, n=297 men) Source: GNHIES98 (1997-1999), DEGS1 (2008-2011), Du et al. [14, 21]

For the indicator HbA1c treatment goal, no substantial differences between the low education group and the medium and high education group were evident for both survey periods. Over time, the proportion of people with type 2 diabetes that achieve the HbA1c goal increased almost equally in all education groups. The patterns and temporal developments were similar for both genders (Figure 4). The same applies for the indicators non-HDL cholesterol goal and blood pressure goal. However, only a small proportion of persons with type 2 diabetes reached the non-HDL cholesterol goal; only men from the medium or high education group achieved a more moderate result (nearly 50%) in the more recent survey period. For the proportion of people with type 2 diabetes who reach the blood pressure goal, the overall increase over time was less pronounced compared to the proportion of those who reached the HbA1c or non-HDL cholesterol goal. As a tendency, a slightly higher proportion of men in the medium and high education group reached the blood pressure goal compared to those in the low education group.

#### Self-management and medical care

The diabetes surveillance system also considers further indicators such as self-monitoring of blood glucose, medical eye and foot examinations as well as taking lipid-lowering statins based on national guidelines for the therapy of type 2 diabetes [16] and the prevention and treatment of retinal and foot complications [22, 23] as well as European recommendations for the prevention of cardiovascular diseases in the presence of diabetes [20] (Info box 5). These indicators reflect results on the quality of diabetes care processes through the respective proportion of type 2 diabetes patients with self-management or medical care.

**Figure 5:**
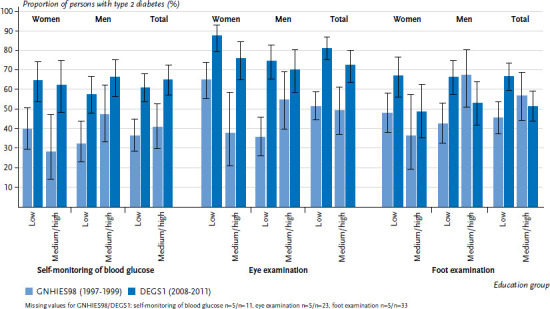
Comparison over time of the proportion of 45- to 79-year-old type 2 diabetes patients with self-monitoring of blood glucose, eye examination and foot examinations according to gender and education level (GNHIES98 n=161 women, n=156 men; DEGS1 n=226 women, n=297 men) Source: GNHIES98 (1997-1999), DEGS1 (2008-2011), Du et al. [14, 21]

For both survey periods, the indicator self-monitoring of blood glucose showed no significant differences between the low education group and the medium and high education group. Over time, the proportion of type 2 diabetes patients who self-monitor their blood glucose level increased considerably, and this increase was similar across the education groups. These findings apply in principle to both genders, whereby the proportion of men in the two upper education groups who self-monitor their blood glucose level is slightly higher in both survey periods compared to the low education group (Figure 5). These observations cannot be fully transferred to the indicators for eye and foot examination. For eye examination in both survey waves, the proportion of women with higher levels of education who have had this examination during the last twelve months tended to be lower compared to those with low education. And the proportion of men with higher levels of education who have had this examination increased to a smaller extend over time than for men with low education. The proportion of persons with type 2 diabetes who have had their feet examined by a doctor during the last twelve months was lower in the higher education groups compared to the low education group for the period 2008 to 2011, and this is related to the increase observed over time in the low education group only.

**Info box 5:**

**Indicators of quality of care for type 2 diabetes – self-management and medical care Self-monitoring of blood glucose:**

**►** Self-monitoring of blood sugar levels

**Eye examination:**

**►** Ophthalmologic examination of the ocular fundus during the last twelve months

**Foot examination:**

**►** Medical examination of the feet during the last twelve months

**Statin use:**

**►** Taking the prescribed statin group medications (cholesterol synthesis-enzyme inhibitors; ATC codes C10AA, C10BA)

Each indicator is given as proportion (in %) of persons with type 2 diabetes who achieve the respective care target in relation to all persons with type 2 diabetes.

Source: Nationale Diabetes-Surveillance am Robert Koch-Institut 20188 [7], Du et al. 2015 [14]

ATC = Anatomical Therapeutic Chemical Classification System

**Info box 6:**

**Health-related quality of life of people with type 2 diabetes**

Based on 36 questions (Short Form-36 Health Survey questionnaire, SF-36) and the scales developed from these on eight dimensions of health, two sum scales are calculated:

**Physical dimension:**

**►** Physical sum scale as the sum of the eight scales, whereby the highest weighting is given to the scales for physical functioning, physical role functioning, bodily pain and general health perception.

**Mental dimension:**

**►** Mental sum scale as the sum of the eight scales, whereby the highest weighting is given to the scales for vitality, social functioning, emotional role functioning and mental health.

Both dimensions of health-related quality of life can potentially achieve values between 0 and 100 and have been transformed for a comparison between GNHIES98 (SF-36V1) and DEGS1 (SF-36V2) into a sample mean of 50 and a standard deviation of 10. This article compares the mean values for different groups of people with type 2 diabetes, whereby a higher mean value represents a higher health-related quality of life.

Source: Nationale Diabetes-Surveillance am Robert Koch-Institut 2018 [7], Ellert et al. 2013 [11]

**Figure 6:**
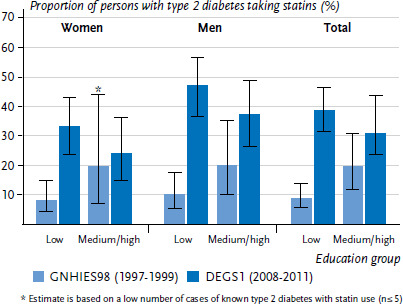
Comparison over time of the proportion of 45- to 79-year-old persons with type 2 diabetes who take statins according to gender and education level (GNHIES98 n=161 women, n=156 men; DEGS1 n=226 women, n=297 men) Source: GNHIES98 (1997-1999), DEGS1 (2008-2011), Du et al. [14, 21]

For the period 1997 to 1999, a higher proportion of type 2 diabetes patients with higher levels of education took statins compared to those with low levels of education, whereas for the period 2008 to 2011, the proportion of those taking statins tended to be higher in the low education group. This result is due to a greater increase in the use of statins in the low education group compared to the higher education groups. These observations apply to both genders (Figure 6).

#### Health-related quality of life

In the diabetes surveillance, subjective assessments are also considered as relevant care indicators. These include self-perceived health-related quality of life, which is obtained by summing up the scales for the physical and mental dimensions of quality of life (Info box 6). In addition to objectively measureable care indicators (such as the treatment goals mentioned above), self-perceived quality of life plays an important role for health and well-being and is therefore anchored as a general therapy objective in the national guideline for the therapy of typ 2 diabetes (‘maintaining or regaining quality of life’) [16].

**Figure 7:**
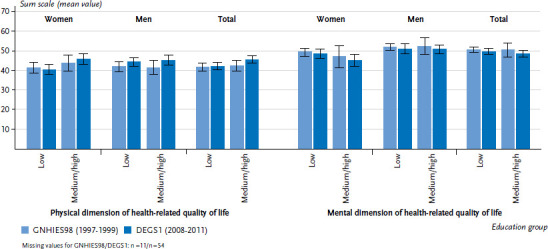
Comparison over time of mean values of sum scales for the physical and mental dimensions of health-related quality of life in 45- to 79-year-old persons with type 2 diabetes according to gender and education level (GNHIES98 n=161 women, n=156 men; DEGS1 n=226 women, n=297 men) Source: GNHIES98 (1997-1999), DEGS1 (2008-2011), Schmidt et al. [24]

Regarding the physical dimension of health-related quality of life for type 2 diabetes patients, the period 1997 to 1999 showed a similar – and the period 2008 to 2011 a slightly more positive – self-assessment in the higher education groups compared to the low education group. This can be traced back to a marginally improved self-assessment over time in the higher education groups. Regarding the self-assessed mental dimension of health-related quality of life, no differences between education groups were present in either survey period and a slight decline over time is apparent. This pattern applies to both genders (Figure 7).

## 4. Conclusion

For public health research and health policy, analyses of trends in health inequality over time are a key factor to develop necessary target group focused prevention measures and evaluate existing prevention programmes.

As the results of this and other studies indicate, higher prevalence of diabetes in the low education group appears to be a persistent issue [12, 25-28]. The prevalence of both known and unknown diabetes remains twice as high in the low education group compared to the medium or high education group [12]. The observed decline of unknown diabetes between the two survey periods, 1997 to 1999 and 2008 to 2011, across all education groups and the simultaneous and roughly equivalent increase of known diabetes could indicate general improvements in the early detection of diabetes (secondary prevention). However, additional measures that aim at reducing the risk of developing diabetes (primary prevention) and focus primarily on the low education group seem necessary.

This fact is highlighted by the results for the 5-year risk of developing type 2 diabetes, which is twice as high for the low education group compared to the medium and high group and has clearly decreased only in the high education group [13]. Studies of the individual behaviour-related risk factors of smoking and physical inactivity indicate a similar widening of inequality due to improvements exclusively in the groups with high education or high professional status [29, 30]. In addition to prevention strategies that aim to promote healthy lifestyles (behavioural prevention), such as those already anchored in the national health target for type 2 diabetes [31], more emphasis needs to be put on the development of living environments and frameworks that promote healthy living (structural prevention) and reach all education groups.

With regard to the diabetes care situation, a positive development has been that improvements over time have been achieved in several areas with no clearly visible differences between education groups. Improvements are not only reflected for several indicators (HbA1c, non-HDL cholesterol and blood pressure treatment goals, blood glucose self-monitoring and eye examination) across different education groups in this study on the basis of RKI survey data [14]. Moreover, further sources providing additional data also indicate improvements in a row of aspects of care. Regional KORA (Cooperative Health Research in the Region of Augsburg) study data, for example, show an increase in the proportion of persons with type 2 diabetes who achieve their treatment goal for HbA1c, blood pressure and LDL cholesterol as well as a decline in the 10-year risk for coronary heart diseases [32]. Current data from studies by the DIAB-CORE (Diabetes-Collaborative Research of Epidemiologic Studies) consortium indicate an increase in awareness, treatment and control of hypertension of people both with and without type 2 diabetes [33]. An earlier analysis of DIAB-CORE data showed that educational level had no influence on the presence of a high blood pressure or lipid metabolism disorders in persons with type 2 diabetes [34]. Furthermore, data from the Disease Management Program (DMP) from the North Rhine region reflect an increase in type 2 diabetes patients who achieve their blood pressure quality targets and have taken part in the recommended diabetes course as well as a stably high proportion of patients with an HbA1c value <8.5%. In 2017, ten out of 14 quantitatively assessable and contractually defined DMP quality targets were achieved [35]. Moreover, data from a regional stroke registry show that while the stroke rate has remained nearly constant for people without diabetes, it has decreased for people with diabetes [36]. National data from hospital statistics (DRG statistic) show a decrease in lower limb amputations (major amputations) in hospitalised diabetes patients [37]. Jointly, these positive trends could indicate improved outpatient care or better self-monitoring of diabetes. These positive developments may also stem from the introduction of modules to the national guideline for the therapy of type 2 diabetes on specific diabetes complications, which encourage diabetes patients to play an active role in their treatment [38] as well as the DMP implementation for type 2 and type 1 diabetes [39]. Analysis of DMP data shows that a continuous DMP participation increases the chance for patients to achieve defined targets for diabetes quality of care [40].

Nonetheless, the currently available study results on care of people with type 2 diabetes all highlight the considerable potential for improvement in the prevention of secondary and concomitant diseases (tertiary prevention). In the present study, for example, the proportions of persons with type 2 diabetes who achieved the non-HDL cholesterol treatment goal or took statins were below 50% across all education groups in the period 2008 to 2011, and less than 70% had their feet examined by a doctor. In spite of the mentioned improvements, KORA and DIAB-CORE data also indicate a suboptimal management of blood sugar levels and of the cardiovascular risk factors of high blood pressure and high LDL cholesterol levels in persons with type 2 diabetes [32, 33]. Moreover, DMP data from the North Rhine region show that only 51% of type 2 diabetes patients with severe foot ulcers also receive treatment from an institution specialized in treating the feet of diabetics, although the agreed target value is at least 75% [40]. In addition, for certain aspects the development over time has stagnated or begun to reverse. In this study, an increase in the proportion of persons with type 2 diabetes who had their feet examined by a doctor and took statins was limited to the low education group. With regard to the physical dimension of quality of life, however, improvements were only seen in the high education group. Irrespective of the education level, persons with type 2 diabetes generally saw a slight deterioration in the mental dimensions of quality of life. Interestingly, an education gradient for the physical dimension of quality of life, with better values achieved by the high education group, was also observed for the 30- to 49-year-old general population based on socioeconomic panel (SOEP) data. This was not, however, observed for the mental dimension [41]. For first-time renal replacement therapy, no decline was observed either in the diabetic or non-diabetic population based on data of a regional dialysis centre [42]. Further, North Rhine DMP data indicate a decline in the proportion of type 2 diabetes patients who have had their kidney function tested during the last twelve months [35]. According to epidemiological studies, the incidence rates of complications such as heart attack, stroke, lower limb amputations, loss of sight and renal insufficiency remains two to eight times higher for people with diabetes than for those without diabetes, despite improvements observed in some of these complications [43].

Moreover, our results and further studies highlight that in addition to educational differences, gender differences also persist with regard to prevalence, risk and care of diabetes. Men, for example, show a higher prevalence of unknown diabetes compared to women [12, 44] as well as a higher risk of type 2 diabetes [45-47]. Regarding treatment, the results of this study show that for women with known diabetes the proportions of those who achieve the non-HDL cholesterol treatment goal or take statins are lower than that of men. Extended analyses based on RKI survey data reflect that gender differences in statin use are no longer significant once a cardiovascular disease is diagnosed in addition to diabetes [21, 48]. This observation points to a less strict tertiary preventive medication therapy of female compared to male diabetes patients. A possible explanation currently under discussion is the higher frequency of the unintended side-effects statins have on women than on men [49]. A further possibility is that diabetes is underrated as a cardiovascular risk factor in women. It has been shown that the cardio-protective effect in women compared to men is significantly reduced by the presence of a diabetes, in particular with regard to lipid metabolism disorders, central obesity and the risk for a heart attack [50-53]. Because cardiovascular diseases in men develop on average around ten years earlier compared to women [50, 51], it is plausible that the proportion of people with cardiovascular comorbidities, including among diabetes patients, is overall considerably higher for men than for women, as is shown, for example, in regional registry and DMP data for the North Rhine region [36, 54, 55].

This article presents selected indicators related to diabetes risk, prevalence and quality of care, which will be continuously presented in the context of the national diabetes surveillance based on RKI survey data. The strengths of this data resource lie in the possibilities to combine interview data with measurement and laboratory data as well as with subjective perceptions, and thereby compare population groups stratified by gender, age or education. For the interpretation of stratified results in this article, it is important to remember that this is a descriptive analysis. Extended complex analyses would be useful to analyse the effects of explanatory factors, such as potential age differences between education groups and possible cohort effects [56], on the observed results. Furthermore, the assessment of social inequality in this article is based exclusively on the operationalisation of individual school education and professional training. Other established operationalisations to describe social inequality of people or households are based on professional status, income or multi-dimensional indices composed of the three dimensions of education, occupational status and income [56, 57]. Moreover, to operationalise social inequality, regional level measures such as the unemployment rate, poverty risk rate or again multi-dimensional indices are often applied, for example, when individual level data are not available [58, 59].

The limitations connected to the applied RKI survey data are that certain groups of people – in particular seriously ill persons, nursing home residents, persons with a migration background, and (in examination surveys) persons aged 80 years and over – have so far not been representatively included and that the limited number of feasible survey participants usually does not allow multi-layered stratifications. For this study, for example, the number of cases following stratification by education group and, additionally, gender were sometimes too small to evidence statistical significance, despite the potential existence of differences between groups.

Looking to the future, a stable data synthesis of key diabetes indicators by merging of RKI survey data with available secondary data, which are discussed in the article Secondary data in diabetes surveillance – co-operation projects and definition of references on the documented prevalence of diabetes in this issue [60], should be ensured in the context of the national diabetes surveillance system [6]. The planned next steps include a regular review of the set of indicators [7] for required adaptions (for example, regarding new available data sources or updating of guideline recommendations) as well as a regular and structured provision of results [61] to promote the planning and evaluation of measures aiming at reducing diabetes risk and supporting early detection and optimal treatment of diabetes. It will thereby remain relevant to consider socioeconomic factors to assess the need, orientation and prevention potential of target group- or settings-focused measures.

## Key statements

- The prevalences of known and unknown diabetes and the 5-year risk of diabetes are considerably higher in the low education group than in the medium or high education group.
- There is no evident shift of the education gradient in the prevalence of known and unknown diabetes over time.
- For the 5-year risk of diabetes, there are indications of a further divergence in the educational gap, which can be attributed to a clear reduction of this risk in the high education group.
- There are no pronounced differences regarding quality of diabetes care between groups with different levels of education.
- Overall, the quality of diabetes care has improved over time.
